# Metastatic Intraparotid Balloon Cell Melanoma: A Diagnostic Challenge

**DOI:** 10.1007/s12105-025-01814-x

**Published:** 2025-07-14

**Authors:** Reed A. McKinney, Kerry B. Baumann, Robert D. Foss

**Affiliations:** 1 Department of Oral and Maxillofacial Pathololgy, Naval Postgraduate Dental School, Bethesda, MD USA; 2 Department of Pathology, Naval Medical Center San Diego, San Diego, CA USA; 3 Joint Pathology Center, 606 Stephen Sitter Ave, Silver Spring, MD 20910-1290 USA

**Keywords:** Melanoma, Metastasis, Parotid gland, Histiocytic, Immunohistochemistry

## Abstract

**Background:**

BCM is an uncommon cytomorphologic variant with the potential to mimic unrelated neoplastic conditions, especially in the absence of a known cutaneous primary lesion or limited access to immunohistochemical stains.

**Case Presentation:**

A 28-year-old male patient presented with recent onset parotid gland mass. The mass was excised, and the subsequent workup elicited an interpretation of metastatic balloon cell melanoma (BCM). Despite an extensive evaluation, no evidence of a primary tumor site was identified.

**Diagnosis:**

The diagnosis was confirmed based on positive staining for BRAF, HMB45, Melan-A, PRAME, S100, and SOX-10 in the malignant cells.

**Conclusion:**

Familiarity with the histologic and immunophenotypic findings may help to ensure an accurate diagnosis.

Balloon cell melanoma (BCM), or melanoma with balloon-cell features is a rare variant with a frequency of roughly 0.15%, and 76 cases reported in the literature between 1970 and 2020 [1, 2]. This melanoma variant is defined by the presence of over 50% ballooned melanocytes. These are large, vacuolated cells with clear or foamy cytoplasm, commonly arranged in sheets. Balloon cell nuclei are typically pleomorphic and hyperchromatic and may contain prominent nucleoli. Expected positive immunostains are comparable to conventional melanoma and include HMB45, Melan-A, S100 and SOX10. Cytokeratin and epithelial membrane antigen (EMA) are characteristically negative [2, 3]. Fontana-Masson stain often fails to reveal pigment [3]. BCM is known to metastasize, with reported spread to the supraclavicular region, scalp, brain, cervical spine, neck, axillary and inguinal lymph nodes, endometrium, as well as in and around the scar of the primary lesion [4, 5]. Balloon cells are usually sparse in the primary melanoma but have the potential to constitute the entire metastatic deposit [3].

An otherwise healthy 28-year-old male presented with a three-month history of an enlarging right parotid mass. The patient reported concurrent flu-like symptoms and possible unplanned weight loss. MRI imaging revealed a circumscribed, T2 hyperintense, T1 isointense to muscle, homogeneously enhancing lesion in the right parotid tail measuring 3.8 cm in greatest dimension (Fig. 1).

**Fig. 1:**
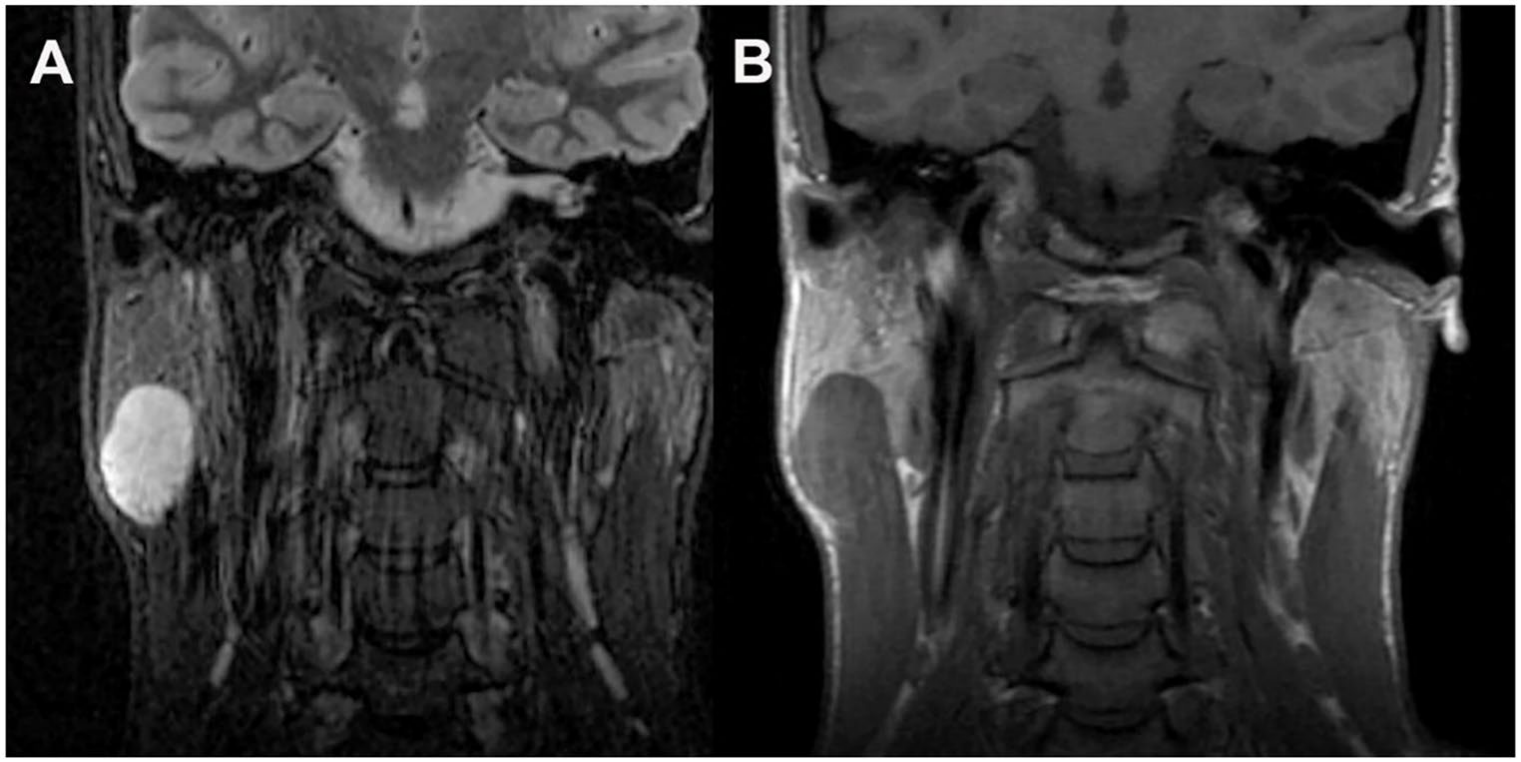
Coronal MR imaging of T2 hyperintense parotid tail mass. **A**) T2 (FSE STIR). **B**) T1

The lesion was excised and consisted of a presumed intra-parotid lymph node extensively effaced by a proliferation of large atypical, histiocytic appearing cells (Fig. 2A). The large polygonal to round cells had voluminous clear to foamy cytoplasm, central pleomorphic nuclei, and occasional prominent nucleoli. Some of the cells had notable nuclear indentations (Fig. 2B). Emperipolesis was not observed in the large cells. A minor subset of cells had an epithelioid appearance with enlarged nuclei and eosinophilic cytoplasm. Admixed lymphoid tissue and interspersed fibrous bands were present.

Immunohistochemical studies revealed the large, atypical cells to be positive for BCL-1 (scattered), BRAF (Fig. 2C), CD68 (Fig. 2D), HMB45, Melan-A, S100, SOX-10 (Fig. 3A-D) and PRAME. Stains for AE1/AE3, ALK-1, CD1a, CD163, GCDFP, p40, and OCT-2 were negative. Subsequently, *BRAF* gene analysis detected the BRAF c.1799T > A (p.V600E) mutation.

**Fig. 2:**
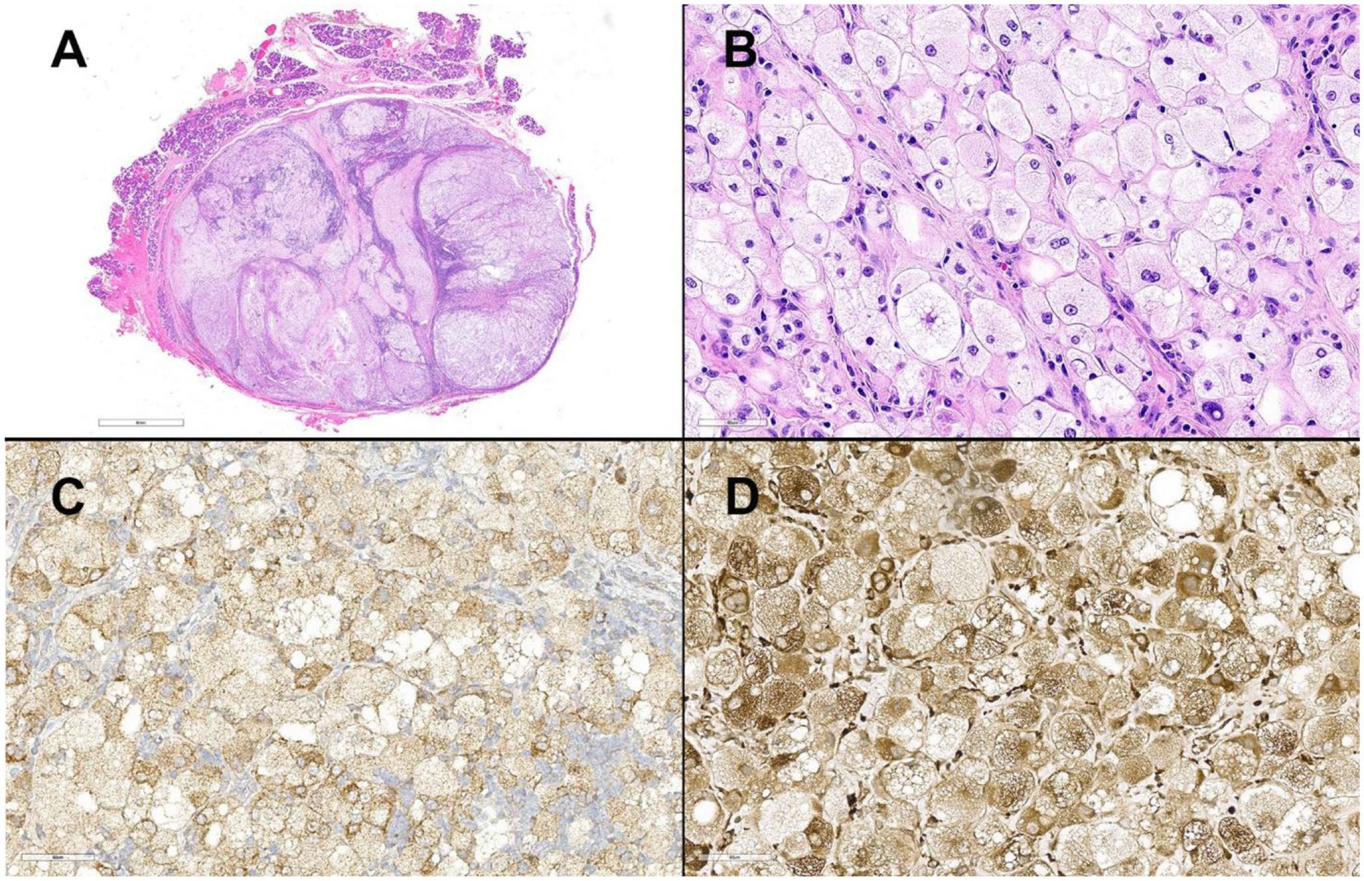
**A**) Scanning power image of the excised mass with parotid tissue at the periphery. **B**) Large round to polygonal cells with vacuolated cytoplasm and central nuclei. These cells are positive for **C**) BRAF and **D**) CD68

**Fig. 3:**
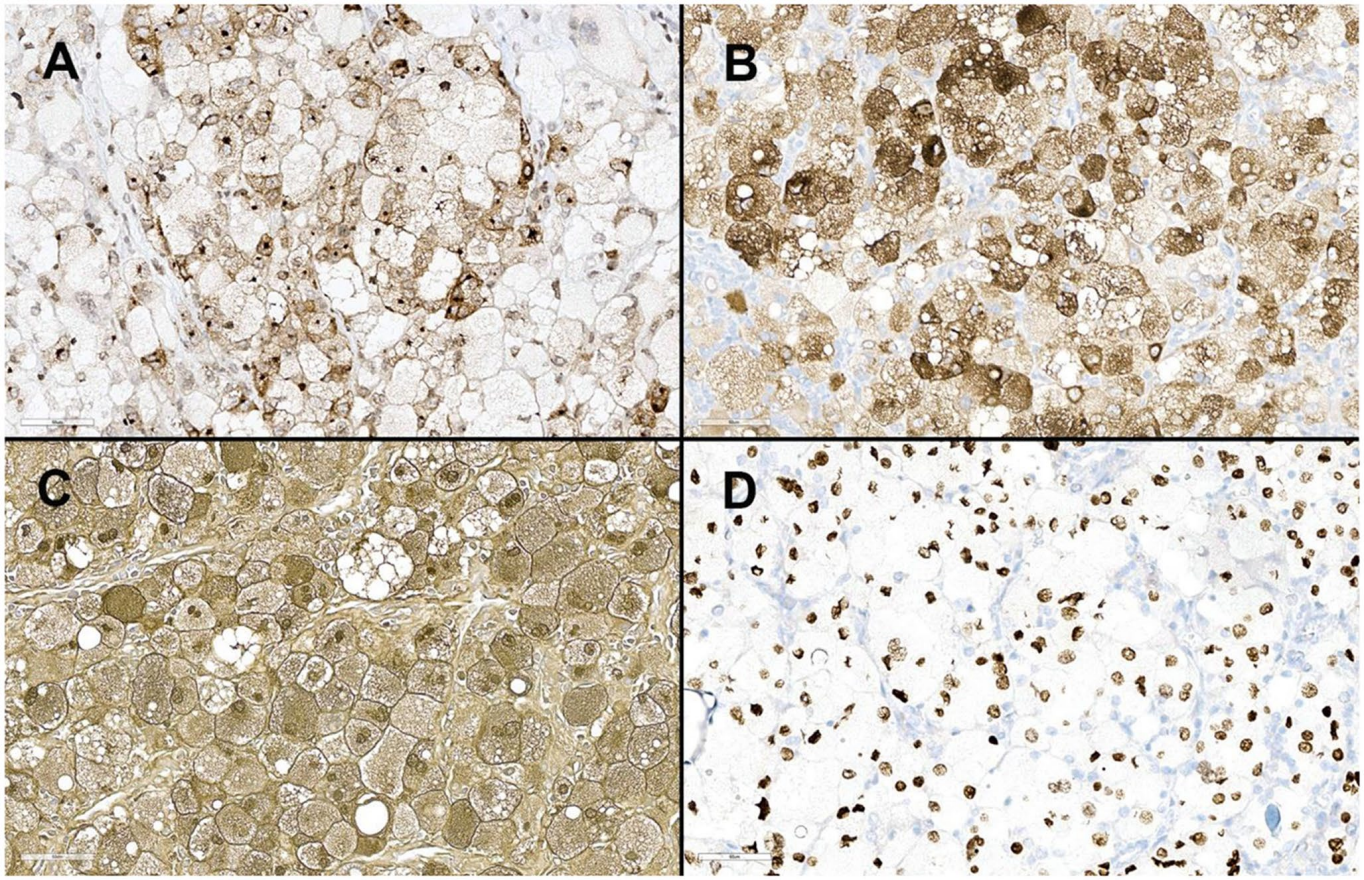
The balloon cells are also positive for **A**) HMB45, **B**) Melan-A, **C**) S100, and **D**) SOX-10

The histologic findings presented a diagnostic challenge, as the cells appeared histiocytic or possibly sebaceous in origin. The initial differential diagnoses included a histiocytic neoplasm, such as Rosai-Dorfman Disease (RDD) or Erdheim-Chester (EC) disease, or a parotid gland sebaceous adenocarcinoma, with melanoma a lesser consideration. In the absence of melanoma specific markers, there was substantial risk of misinterpretation as a histiocytic neoplasm as BCL-1, CD68, S100, and, rarely, BRAF expression is potentially supportive of RDD, while BRAF and CD68 are frequently seen in EC. The absence of emperipolesis and negative OCT-2 staining did militate against an interpretation of RDD. Positive HMB-45, Melan-A, PRAME and SOX-10 stains were confirmatory of metastatic melanoma.

After extensive clinical and imaging correlation, no known primary source of the melanoma was identified. The patient did have a remote (childhood) history of excisional biopsy with skin grafting in the region of the right ear.

## Data Availability

No datasets were generated or analysed during the current study.

## Declarations

### Disclaimer

The opinions and assertions expressed herein are those of the author(s) and do not reflect the official policy or position of the Uniformed Services University of the Health Sciences, the United States Department of the Navy, or the United States Department of Defense. References to non-Federal entities or products do not constitute or imply a Department of Defense, Department of the Navy, or Uniformed Services University of the Health Sciences endorsement.
